# Co-Expression Network Analysis of the Transcriptome Identified Hub Genes and Pathways Responding to Saline–Alkaline Stress in *Sorghum bicolor* L.

**DOI:** 10.3390/ijms242316831

**Published:** 2023-11-27

**Authors:** Hongcheng Wang, Lvlan Ye, Lizhou Zhou, Junxing Yu, Biao Pang, Dan Zuo, Lei Gu, Bin Zhu, Xuye Du, Huinan Wang

**Affiliations:** School of Life Sciences, Guizhou Normal University, Guiyang 550025, China; besthongcheng@163.com (H.W.); 222100100456@gznu.edu.cn (L.Y.); 21010100413@gznu.edu.cn (L.Z.); yujunxing@gznu.edu.cn (J.Y.); 21010100406@gznu.edu.cn (B.P.); 21010100449@gznu.edu.cn (D.Z.); 201808009@gznu.edu.cn (L.G.); zhugg130@126.com (B.Z.)

**Keywords:** saline–alkaline stress, RNA-seq, hormone signal transduction, hub genes

## Abstract

Soil salinization, an intractable problem, is becoming increasingly serious and threatening fragile natural ecosystems and even the security of human food supplies. Sorghum (*Sorghum bicolor* L.) is one of the main crops growing in salinized soil. However, the tolerance mechanisms of sorghum to saline–alkaline soil are still ambiguous. In this study, RNA sequencing was carried out to explore the gene expression profiles of sorghum treated with sodium bicarbonate (150 mM, pH = 8.0, treated for 0, 6, 12 and 24 h). The results show that 6045, 5122, 6804, 7978, 8080 and 12,899 differentially expressed genes (DEGs) were detected in shoots and roots after 6, 12 and 24 h treatments, respectively. GO, KEGG and weighted gene co-expression analyses indicate that the DEGs generated by saline–alkaline stress were primarily enriched in plant hormone signal transduction, the MAPK signaling pathway, starch and sucrose metabolism, glutathione metabolism and phenylpropanoid biosynthesis. Key pathway and hub genes (*TPP1*, *WRKY61*, *YSL1* and *NHX7*) are mainly related to intracellular ion transport and lignin synthesis. The molecular and physiological regulation processes of saline–alkali-tolerant sorghum are shown by these results, which also provide useful knowledge for improving sorghum yield and quality under saline–alkaline conditions.

## 1. Introduction

Due to excessive reclamation by human beings, significant reductions in vegetation, residual salt after the evaporation of soil moisture in irrigated areas [1], and soil drought caused by the continuous warming of the climate in recent years, the problems caused by salinized soil are becoming increasingly intractable and serious. Plants rely on normal saline–alkaline content to maintain regular physiological functions [2]. However, excessive saline–alkali in soil has many adverse effects on plant growth, which is the main environmental stress factor and has been the primary limitation on global agricultural crop production [3,4]. Furthermore, according to the poor distribution of plants, salinization of soil seriously threatens fragile natural ecosystems and even the security of human food supplies [5,6].

The effects of salinized soil on crop growth are not only reflected in salinization but also in alkalinity [7]. Three categories will be set up, based on salt ion concentration and pH value, to express saline–alkaline stress clearly: light (salt < 3%, pH 7.1–8.5), medium (salt = 3%, pH 8.5–9.5), and serious (salt > 6%, pH > 9.5) [8,9]. Stress from high concentrations of salt generally triggers excessive ion poisoning and osmotic stress, which further causes oxidative stress in plants [10]. It is critical that the balance of Na^+^/K^+^ is fundamental for plants’ survival when stressed by external environmental factors [11,12]. Since the mechanisms of absorption of these two cations in plants are roughly the same, a competitive relationship between their absorption exists [13]. Facing salt stress, the absorption of K^+^ will be badly affected in plants owing to the accumulation of Na^+^. The imbalance in the Na^+^/K^+^ ratio within plants impedes the synthesis of specific enzymes, resulting in ion toxicity. For instance, this imbalance can impact the activity of phosphoenolpyruvate carboxylase (PEPC), thus hindering photosynthesis [14]. Moreover, it can suppress nitrate reductase (NR) activity, affecting the nitrogen metabolism pathway [15]. At the same time, a large amount of Na^+^ accumulation will reduce the absorption of the second messenger Ca^2+^ by plants and interfere with plant signal transduction to a certain extent [16,17]. The main driving force of plant water absorption comes from osmotic pressure, of which the reasonable range is the basis of normal plant survival. But a situation occurs when plants are stressed by salt where the osmotic pressure in plant cells and soil inverts accordingly, and abnormal osmotic pressure further leads to water loss in the plants, expansion of the leaf area and closure of the stomata on the plant epidermis, eventually leading to a photosynthetic efficiency decrease and growth suppression in plants [18]. Additionally, when plants suffer from damage caused by ion imbalance, reactive oxygen species (ROS) emerge in large numbers and disrupt the equilibrium, causing programmed cell death and subsequently decreasing plant productivity [19,20]. Typically, soil alkali stress is mostly caused by an increase in NaHCO_3_ and NaCO_3_, which mainly augment the pH value based on salt stress, and a high pH value will seriously disturb the stability of cells and destroy the integrity of the cell membrane [21,22], further affecting ion absorption [23] and causing greater damage to plants [24]. Hence, it is important for ecology and the economy to find crops including superior varieties that are suitable for saline–alkaline soil growth [25].

Sorghum, as a diploid C4 crop, features a well-developed root system and demonstrates high nutrient utilization efficiency [26], resulting in amplified biological and economic yields. It exhibits robust growth even in arid, infertile, and saline–alkali conditions. This versatile crop finds applications in food, fodder, biofuels, and brewing, serving as a dietary mainstay for over 500 million of the world’s impoverished population [27,28,29]. The expansion of saline–alkali territories progressively encroaches upon significant resources [30]. Consequently, probing the molecular mechanisms underlying sorghum’s tolerance to salt–alkali conditions could potentially steer novel research directions to mitigate food crises. Transcriptomics, a pivotal approach in contemporary molecular studies, furnishes a comprehensive understanding of a plant’s transcriptome and its temporal gene expression modifications under stress, facilitating the identification of stress-responsive genes [31,32]. Application of the weighted gene co-expression network analysis (WGCNA) method aids in discerning gene expression patterns across diverse conditions, thereby identifying co-regulated genes and associating them with transcriptomic data to unravel plants’ responses to abiotic stress [33].

Present research concerning the molecular mechanism of salt–alkali tolerance in sorghum primarily concentrates on long-term and multi-stress regulatory mechanisms. However, investigations of the molecular regulatory network during the seedling stage of salt–alkali tolerance are scarce. Consequently, this study endeavors to scrutinize the physiological, biochemical, and transcriptomic data of post-stressed seedling-stage sorghum. The utilization of WGCNA and protein–protein interaction (PPI) analysis aims to screen pivotal genes and significant pathways, thereby revealing the molecular regulatory pathways linked to sorghum’s salt–alkali stress tolerance and adaptation.

## 2. Results

### 2.1. Phenotype of Sorghum under NaHCO_3_ Stress

**Figure 1:**
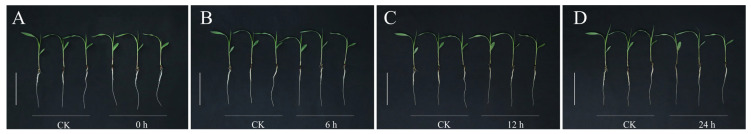
Hydroponic phenotype of sorghum seedlings under 150 mM NaHCO_3_. The left side represents the control check (CK), and the right side represents the treatment group. (**A**) Phenotypes of the CK and the treatment group at 0 h. (**B**) Phenotypes of the CK and the treatment group at 6 h. (**C**) Phenotypes of the CK and the treatment group at 12 h. (**D**) Phenotypes of the CK and the treatment group at 24 h. Scale bar = 7.5 cm.

In general, when sorghum was exposed to 150 mM/L NaHCO_3_, the root length and leaf length of the sorghum did not change significantly every time (Figure 1). The shoot growth rates of the two groups were different, and the shoot length in the control group was slightly but not significantly higher than in the treatment group. This result showed that NaHCO_3_ stress did not have a significant effect on sorghum within 24 h (Tables S1 and S2).

### 2.2. Antioxidant System Responds to Saline–Alkali Stress

**Figure 2:**
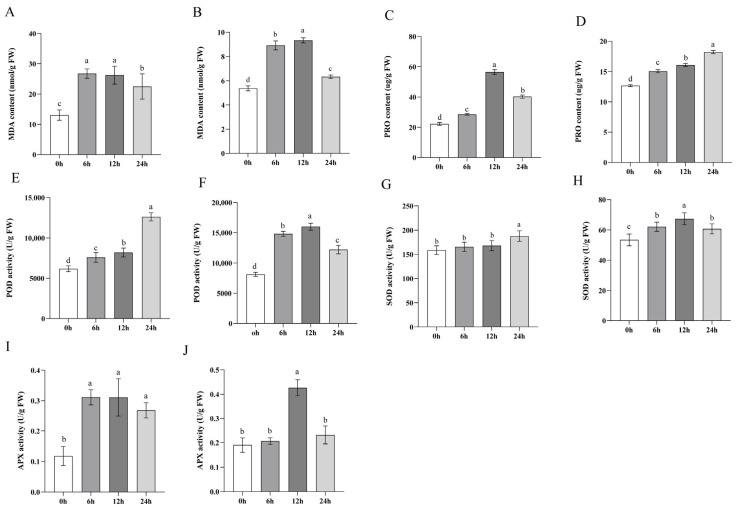
Changes in the antioxidant enzyme activities of sorghum treated with 150 mM/L NaHCO_3_ at different times. (**A**,**C**,**E**,**G**,**I)** represent the changes in MDA, PRO, POD, SOD and APX in the shoots and (**B**,**D**,**F**,**H**,**J**) represent the changes in MDA, PRO, POD, SOD and APX in the roots. There were significant differences among different letter groups (*p* < 0.05).

Under abiotic stress, the increase in ROS contents, such as hydrogen peroxide, is greater than that under normal conditions. In plants, recovery from stress injury depends on the resilience of the antioxidant system, including antioxidant enzymes and nonenzymatic substances [34]. Therefore, to detect the physiological response of sorghum to NaHCO_3_ stress, the contents of nonenzymatic antioxidants (MDA and PRO) and the activities of antioxidant enzymes (APX, SOD and POD) were measured at different times after treatment with NaHCO_3_. The data suggest a temporal fluctuation in malondialdehyde (MDA) levels, which serves as a marker for cellular damage. Both root and shoot samples demonstrate an initial increase in MDA, followed by a decline. The content of PRO in the shoots serves as a crucial indicator measuring plant stress tolerance; the levels of PRO exhibit variation between the aboveground and belowground parts. In response to stress, POD significantly mitigates cellular damage caused by reactive oxygen species through peroxide degradation, and the same function is performed by SOD and APX. In the shoots, MDA and APX peaked at 6 h, at 2.25 and 2.53 times that in control group (Figure 2A,I), PRO peaked at 12 h, at 2.64 times that in control group (Figure 2C), while POD and SOD content steadily rose and peaked at 24 h, at 2.08 and 1.09 times that in control group (Figure 2E,G). In the roots, MDA, POD, SOD, and APX in the roots reached the highest values at 12 h and then decreased, with increases of 78%, 100%, 18% and 121%, respectively, compared to control plants (Figure 2B,F,H,J), while PRO in the roots reached its highest value at 24 h, 40% higher than in the control group (Figure 2D). 

### 2.3. Statistics of DEGs from RNA-Seq Data

**Figure 3:**
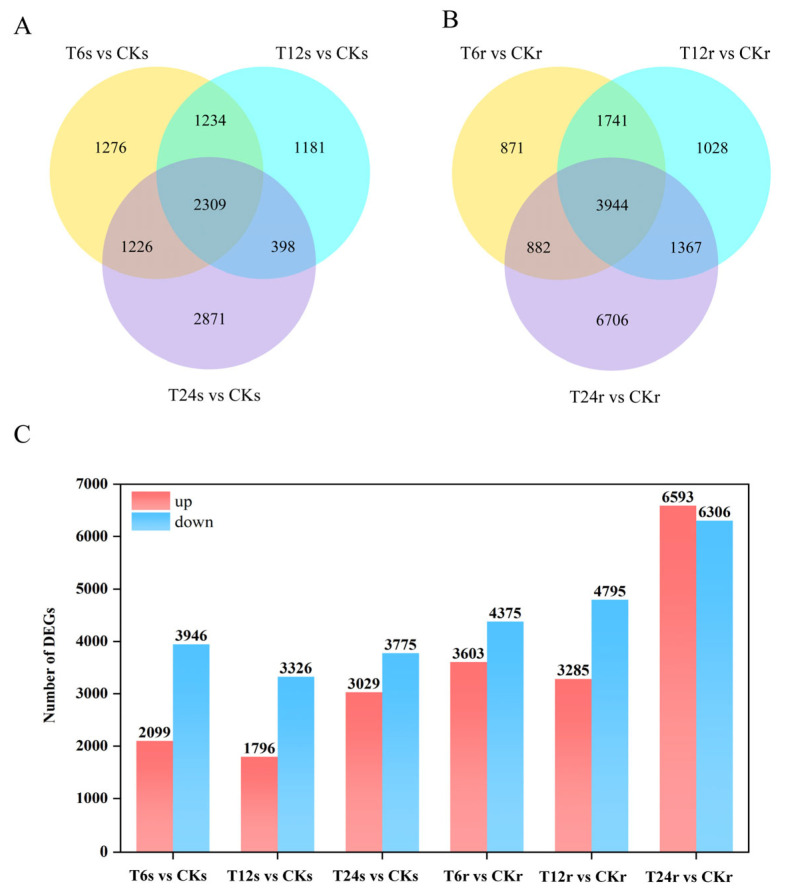
DEGs in sorghum exposed to saline–alkaline. Venn diagram of DEGs that were unique to and exposed to saline–alkaline. (**A**) Venn diagram of DEGs in shoots in control and saline–alkaline for 6, 12 and 24 h; (**B**) Venn diagram of DEGs in roots in control and saline–alkaline for 6, 12 and 24 h; (**C**) Numbers of up- and downregulated genes in sorghum exposed to saline–alkaline (T6s vs. CKs, T12s vs. CKs, T24s vs. CKs, T6r vs. CKr, T12r vs. CKr and T24r vs. CKr).

After 0, 6, 12 and 24 h, DEGs (between different groups shared by all measured time points) were screened for different treatment groups. In the shoots, 6045, 5122 and 6804 DEGs were detected in T6s vs. CKs, T12s vs. CKs, and T24s vs. CKs individually (where CK stands for the control group treated at 0 h) (Figure 3A). Among these, 2099, 1796 and 3029 genes were essentially upregulated, and 3946, 3326 and 3775 genes were significantly downregulated. In the roots, 7978, 8080 and 12,899 genes were detected in T6r vs. CKr, T12r vs. CKr, and T24r vs. CKr (Figure 3B), respectively. Totals of 3603, 3285 and 6593 genes presented significantly higher expression levels, and 4375, 4795 and 6306 genes were expressed at significantly lower levels. The analysis of DEGs between different groups showed that 2309 DEGs were detected in shoots and 3944 DEGs in roots overall (Figure 3C).

### 2.4. Biological Function Analysis of DEGs Corresponding to Saline–Alkaline Stress

**Figure 4:**
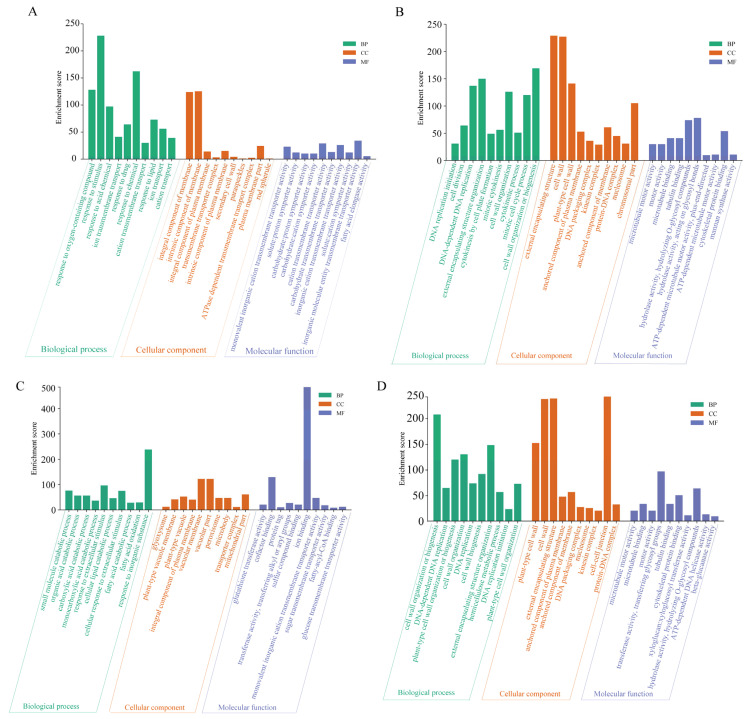
GO analysis of sorghum exposed to saline–alkaline. Upregulated DEG enrichment in shoots and roots (**A**,**C**); enrichment of shoots and roots for downregulated DEGs (**B**,**D**).

For the upregulated and downregulated genes, gene ontology (GO) was employed for functional enrichment analysis (Figure 4). The results showed that the upregulated GO terms in shoots and roots were mainly enriched in the organelle membrane (Figure 4A,C), potassium ion transmembrane transport, ion transmembrane transporter activity, response to acid chemicals, and other items. This shows that the cell membrane will perceive and resist saline–alkaline stress and produce osmotic adjustment substances to maintain the homeostasis of the cell with increasing stress duration [35]. Numerous plants possess the capacity to excrete a substantial quantity of organic acids for regulating intracellular pH and balancing ions when subjected to saline–alkali stress. This process aids plants in fortifying their resilience against unfavorable environmental conditions [36]. The downregulated DEGs were mainly enriched in microtubule motor activity (Figure 4B,D), anchored components of the plasma membrane, cellular component organization or biogenesis, DNA replication initiation, and other items. Saline–alkaline stress could lead to a decrease in the accumulation of plant biomass.

**Figure 5:**
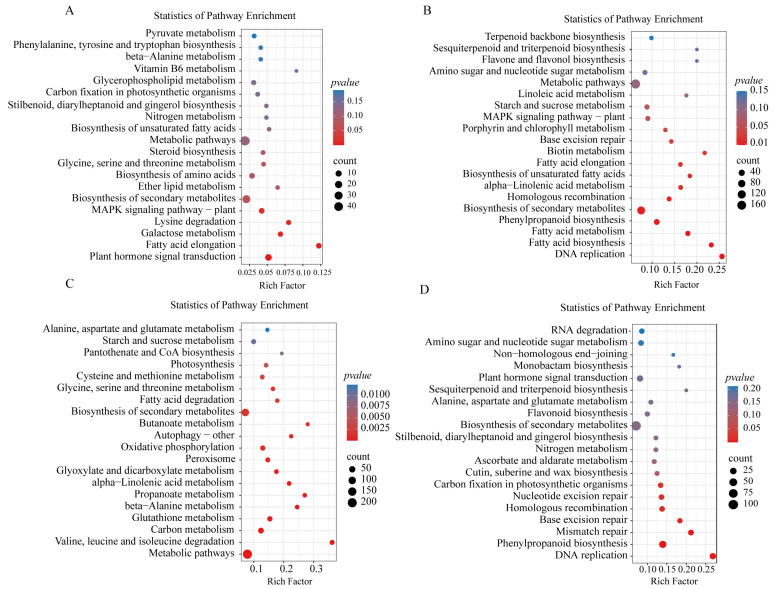
TOP 20 KEGG pathway enrichment analysis of significant DEGs. Upregulated DEG enrichment in shoots and roots (**A**,**C**); enrichment of shoots and roots for downregulated DEGs (**B**,**D**).

To explore the impact of salt–alkali stress on sorghum, an analysis of KEGG enrichment pathways was undertaken (Figure 5). In the aerial parts of sorghum subjected to salt–alkali stress, 14 DEGs were associated with plant hormone signal transduction, while 29 DEGs were involved in the biosynthesis of secondary metabolites. Among the downregulated genes, 30 DEGs were associated with phenylalanine metabolism, with 16 DEGs linked to starch and sucrose metabolism. Within the roots of salt–alkali-stressed sorghum, the upregulated genes demonstrated the involvement of 22 DEGs in glutathione metabolism and 12 genes in beta-alanine metabolism. Among the downregulated genes, 14 DEGs were associated with mismatch repair, 38 DEGs with phenylalanine metabolism, and 12 DEGs with nucleotide excision repair.

### 2.5. Construction of WGCNA and Biological Function Analysis

**Figure 6:**
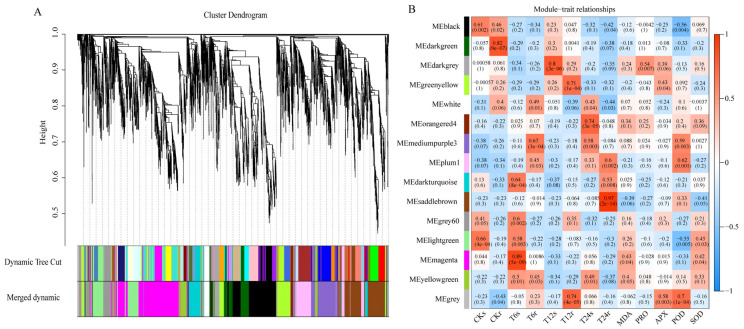
(**A**) Clustering dendrograms of genes regulated to saline–alkaline stress. The 14 co-expression modules are shown in different colors. (**B**) Module–trait associations under saline–alkaline stress. The colors, ranging from blue through white to red, indicate low to high correlations.

After culling low-expressed genes from the sorghum expression matrix, specifically genes with FPKM < 1, a total of 15,912 DEGs were delineated. To identify potential gene modules associated with salt–alkali stress, a WGCNA was executed on the filtered DEGs. This analysis led to the detection of 14 distinctive gene modules, each distinguished by a unique color scheme (Figure 6). Post an examination of positive correlation coefficients, three modules demonstrating the most robust positive correlations were singled out. These three modules exhibited pronounced relevance to NaHCO_3_ stress and were denoted as magenta, darkgrey, and saddlebrown. Functional enrichment analyses encompassing GO and KEGG were then conducted on these three co-expressed modules. The GO enrichment analysis revealed primary enrichment of the DEGs in catalytic activity and metabolic processes (Table S3). Additionally, the KEGG enrichment analysis indicated a significant enrichment of the mitogen-activated protein kinase (MAPK) signaling pathway, starch and sucrose metabolism, and glutathione metabolism, as well as plant hormone signal transduction within these modules (Table S4).

### 2.6. PPI Network Validated the Key Hub Genes

**Figure 7:**
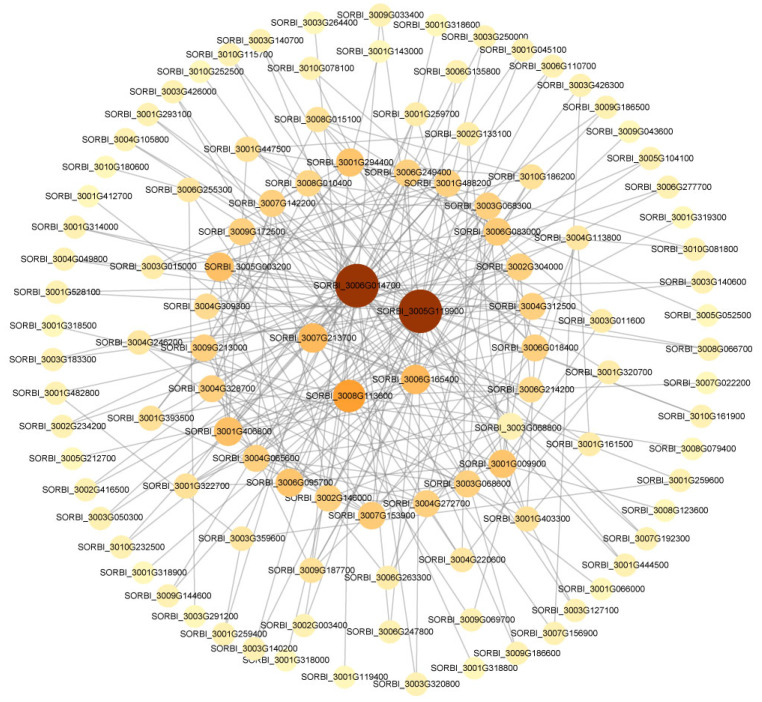
The protein–protein interaction (PPI) network associated with DEGs in shoot and root co-response under saline–alkaline stress. The nodes are proteins and the links are interactions between them.

The analysis conducted through WGCNA under saline–alkaline stress revealed significant pathways, such as the MAPK signaling pathway, starch and sucrose metabolism, plant hormone signal transduction, phenylpropanoid biosynthesis, and glutathione metabolism. Differentially expressed genes within these pathways were selected to construct a protein–protein interaction (PPI) network (Figure 7). The final set of identified hub genes primarily encompassed cinnamyl alcohol dehydrogenase and glutathione synthetase (Table S5). Enrichment analysis demonstrated the interplay among these proteins within the group functions.

### 2.7. Identification of DEGs Encoding Transcription Factors

**Figure 8:**
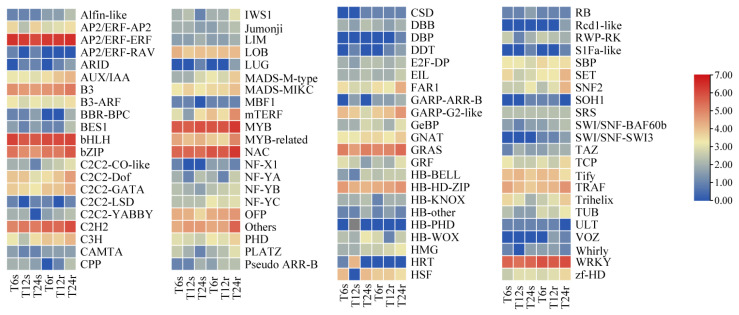
Heatmap of quantitative changes in transcription factors in different time periods.

Under saline–alkaline stress, transcription factors (TFs) can receive upstream signals and regulate downstream gene expression in response to stress [37]. According to the study, 85 transcription factor families were predicted (Figure 8). The largest number of family members was the AP2/ERF-ERF family, with a total of 127, followed by basic helix–loop–helix (bHLH), MYB, NAM, ATAF1/2, CUC2 (NAC), WRKY and other TF families. Most of the DEGs encoding AP2/EFR–ERF, bHLH, MYB and WRKY transcription factor families were upregulated, while those encoding NAC and C_2_H_2_ TF families were mostly downregulated. We performed an analysis of the top five DEGs encoding TFs with large differential expression folds (Table 1). Among them, the transcription factors with the largest differential fold changes in AP2/ERF, bHLH and MYB, and NAC and WRKY were SORBI_3002G060400, *AIG1* (SORBI_3009G057000) and *MYB3R-4* (SORBI_3001G056000), respectively.

### 2.8. Analysis of DEGs Involved in Phytohormone Biosynthesis and Signal Transduction

**Figure 9:**
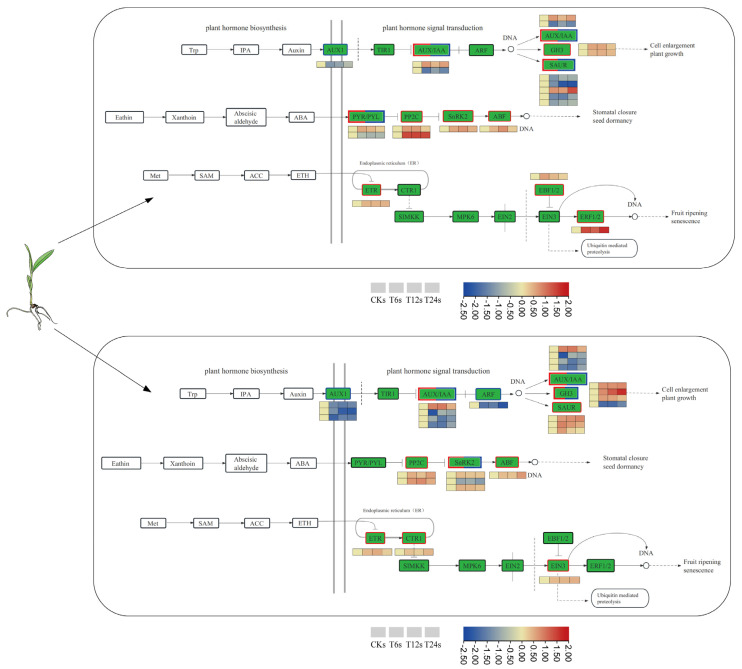
Transcriptional changes in DEGs involved in plant hormone biosynthesis and plant hormone signal transduction in shoots and roots. The heat maps from left to right are CKs, T6s, T12s and T24s in leaves and CKr, T6r, T12r and T24r in roots. Different colors represent different multiples of difference.

With saline–alkaline stress, DEGs in the shoots and roots were enriched in plant hormone signal transduction pathways for which functional classification was carried out (Figure 9; Tables S6 and S7). Three signal transduction pathways were mainly involved: auxin, abscisic acid (ABA) and ethylene (ETH). The three early auxin response families in auxin signal transduction are auxin/indole-3-acetic acid (AUX/IAA), Gretchen Hagen3 (GH3) and small auxin-up RNA (SAUR). There were 9 DEGs encoding auxin proteins that were upregulated, and 13 were downregulated. Most of the DEGs encoding the AUX/IAA protein were downregulated, and most of the DEGs encoding the GH3 protein were upregulated. However, SAUR displayed a relatively complex regulation of gene expression, since DEGs encoding the SAUR protein in roots were all upregulated and those in shoots showed an upward trend. Interestingly, one differentially expressed gene encoding the ABA-responsive element binding factor (ABF) transcription factor, belonging to the bZIP family, was upregulated both in shoots and roots and may have a large influence on plants stressed by saline–alkaline. In ETH signal transduction, DEGs encoding ethylene-responsive factor 1/2 (EBF1/2) were upregulated in shoots.

### 2.9. Analysis of DEGs Involved in Intracellular Ion Transport

Under salinization conditions, the concentration of Na^+^ changes significantly, and plants activate Ca^2+^ channels when they sense the alteration [38]. Meanwhile, to maintain Na^+^ homeostasis, Na^+^ passively enters the root cell via the K^+^ channel and is evenly transported from the root to the shoot by transporters [39]. In this study, we found 48 DEGs involved in ion channels and transporters in roots and 21 DEGs in shoots (Tables S8 and S9), which indicated that the root presented more DEGs when directly exposure to saline–alkaline stress. A total of 12 genes encoding ion channel proteins, such as calcium-dependent protein kinase (CDPK), calcineurin B-like protein (CBL) and CBL-interacting protein kinase (CIPK), were detected in roots, with 7 DEGs in shoots encoding similar proteins. Moreover, six DEGs encoding potassium transporter (KT) proteins were upregulated in roots, with three DEGs upregulated in shoots at the same time. Six genes encoding ABC transporters were found in shoots, of which four DEGs were upregulated and the remaining two were downregulated.

### 2.10. Validation of RNA-Seq Via RT-qPCR

**Figure 10:**
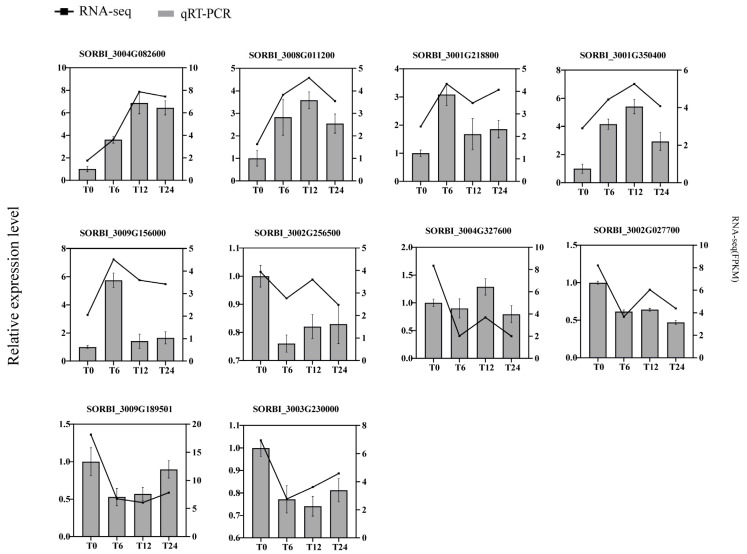
Genetic validation of RNA-seq was performed using qPCR. This validation encompassed five upregulated genes and five downregulated genes. The left *y*-axis denotes the relative expression level, while the right *y*-axis represents RNA-seq (FPKM).

**Figure 11:**
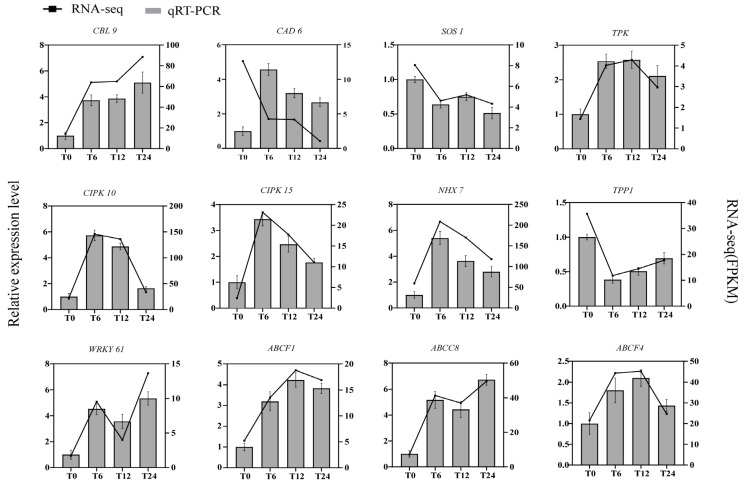
Verification of the expression of pathway genes. The left *y*-axis denotes the relative expression level, while the right *y*-axis represents RNA-seq (FPKM).

To check the accuracy of transcriptome data, we selected 10 genes randomly for RT-qPCR verification (Figure 10); the same method was also used to verify the 12 genes mentioned below in the discussion (Figure 11). T0 was the control group, and T6, T12 and T24 were the treatment groups. The annotation information and primer sequences are listed in the attached files (Table S10). Figure 10 clearly displays that the gene expression trends of the treatment group and the control group were basically consistent, which was followed by the transcriptome gene sequencing results. This section’s results verify the reliability of our data. 

## 3. Discussion

Currently, soil salinization poses a serious menace to agriculture, regional economies and the environment [4]. Saline–alkaline stress directly impacts plants’ hormone balance, photosynthesis and respiration. It also indirectly disrupts the synthesis of macromolecules such as lipids, proteins and nucleic acids, impairing normal physiological processes and limiting crop yield [40,41]. Recently, emerging studies have used transcriptome techniques to elucidate the molecular mechanisms of plant responses to abiotic stress [42,43]. 

Under saline–alkaline stress, plants modulate the expression of related genes and transporters, implementing specific strategies for saline–alkaline adaptation at the cellular level [44]. Additionally, these genes enhance plant resistance through various metabolic pathways, although the duration of stress can also influence pathway regulation [45,46]. Analysis of GO and KEGG enrichment for T6s vs. CKs DEGs revealed that, although the number of upregulated and downregulated DEGs was comparable (Figure 4 and Figure 5), the total number of downregulated genes (3096) exceeded that of upregulated genes (2099). The GO terms primarily encompassed biological processes, cellular processes and responses to stimuli. The KEGG analysis indicated enrichment of the biosynthesis of secondary metabolites and photosynthesis, among other things. This study suggests that after 6 h of treatment, the expression of certain genes involved in maintaining normal physiological activities in plants is inhibited. Upon analyzing the differentially expressed genes in T12s vs. CKs, a reduction of 923 DEGs was observed, with a decrease in 303 upregulated genes and 620 downregulated genes. However, the GO and KEGG enrichment profiles did not exhibit significant changes and were similar to the enrichment patterns observed in T6s. This might be attributed to the rapid expression of certain response genes in plants following a brief period of stress, activating the mechanism for abiotic stress resistance and the re-expression of genes previously inhibited to maintain physiological activities in T6s. Therefore, this study postulates that after 6 h of treatment, the expression of specific normal genes in sorghum shoots was suppressed, while most salt-tolerant genes were not fully activated during this timeframe. After 12 h of treatment, response genes involved in signal transduction and hormone responses were activated, facilitating the implementation of the plant’s resistance mechanism in the roots. As the principal organ of plants [28], roots are also the first to perceive and adapt to saline–alkaline stress [47]. Our findings demonstrate that differentially expressed genes in the roots exhibit a more pronounced increase, with a greater number of DEGs compared with the leaves, indicating a heightened response of the roots to saline–alkaline stress [44] and a more intricate regulatory mechanism and pathway coordination [48].

Plant hormones have long been recognized for their involvement in regulating plant development and tolerance to various stresses [49]. Several plant hormones, including ABA, ETH and IAA, have demonstrated responsiveness to environmental cues [50,51]. ABA, an important plant hormone, plays a crucial role as a signal during the response to saline–alkaline stress [20]. The abscisic acid receptor PYR/PYL senses ABA and forms a complex with it, subsequently binding to the PP2C protein, leading to the release of SnRK2 protein kinases [52]. The activation of this complex activates the downstream transcription factor ABF, positively regulating the ABA signal transduction pathway [53] in response to plant abiotic stress. Ethylene, as a biologically active gaseous hormone, also plays a vital role in plant development and abiotic stress responses. In the ETH signal transduction pathway, ethylene responsive factor (ERF), an ethylene response element, regulates the expression of ethylene response genes [54]. EIN3, a nuclear protein family unique to plants in the ethylene signaling pathway, is also instrumental in regulating ETH signal transduction. Auxin, the earliest discovered plant hormone, exhibits adaptability to the environment, and several gene families participate in auxin signal transduction under saline–alkaline stress, including GH3, SAUR and AUX/IAA [55,56]. AUX1 binds to T1R1, leading to ubiquitination and degradation of AUX/IAA [57]. Saline–alkaline stress induces the expression of the transcription factor ARF, which regulates local biosynthesis [58], while many ARFs are significantly downregulated in roots under salt stress [59]. Our GO analysis revealed that DEGs in the shoots and roots of sorghum were highly enriched in plant hormone signal transduction pathways under saline–alkaline stress (Tables S5 and S6). Furthermore, we constructed a model depicting the response of phytohormone pathways to saline–alkaline stress in sorghum (Figure 10). Our results demonstrate that under saline–alkaline stress, DEGs related to the ABA hormone pathway, such as *PYL8* (SORBI_3004G113800), *TRAB1* (SORBI_3002G225100) and *PP2C51* (SORBI_3009G238600), and the ETH hormone pathway, such as *ERS2* (SORBI_3009G050400) and CTR1 (SORBI_3002G133400), were upregulated. However, two genes, *PYL4* (SORBI_3001G403300) and *SAPK7* (SORBI_3006G083000), were exceptions. DEGs in the IAA hormone pathway were mostly downregulated, including *AUX2* (SORBI_3001G439000), *IAA16* (SORBI_3009G069700) and *ARG7* (SORBI_3006G252466), among others. Additionally, many stress-related genes were associated with plant hormone biosynthesis and signal transduction, potentially contributing to the growth improvement of sorghum under saline–alkaline stress. Previous studies have indicated that overexpression of *TERF1* in tobacco and rice enhances salt tolerance [60,61], and overexpression of *EIN3* in Arabidopsis improves salt tolerance in plants [62]. In our study, we observed an upwards trend in the genes encoding *ERF1B* (SORBI_3001G002500) and *EIN3-like4* (SORBI_3004G189100) in sorghum, suggesting their potential role in enhancing salt tolerance. Similarly, we identified a gene *AUX11* (SORBI_3006G255300) encoding ARF in the root that exhibited the same trend.

**Figure 12:**
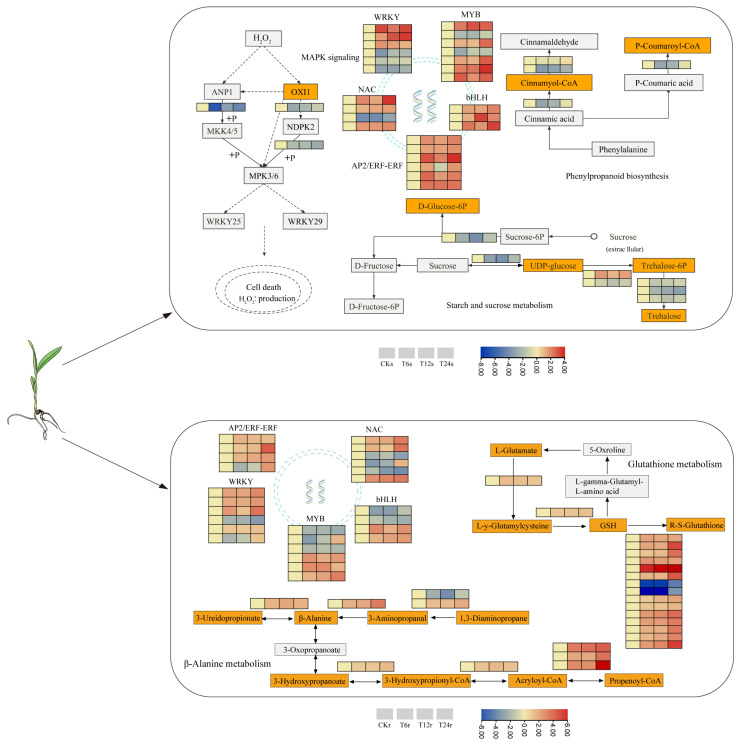
Transcriptome network in shoots and roots. The heat maps from left to right are CKs, T6s, T12s and T24s in shoots and CKr, T6r, T12r and T24r in roots.

After exposure to abiotic stress, plants adjust their metabolic pathways to enhance the tolerance to stressors [63]. This investigation demonstrates that sorghum seedlings improve their resilience to saline–alkaline stress by upregulating the expression of diverse metabolic pathway genes in both the aerial and underground parts. Analysis of KEGG enrichment entries for DEGs in the subterranean region and the correlation module from WGCNA revealed the predominant enhancement of glutathione metabolism and beta-alanine metabolism in root cells to combat saline–alkali stress. Glutathione, an indispensable antioxidant pathway in plant organisms, effectively eradicates reactive oxygen species within cells, which is crucial for preserving cellular redox homeostasis, which is vital in the plant’s defense against stressors [64]. Within this research, the *GST* gene, an essential enzyme for GSH synthesis in glutathione metabolism, exhibited a noteworthy upsurge under saline–alkali stress in the subterranean area [65]. Numerous studies have underscored *GSTs* participation in diverse plant activities and its pivotal role in the plant’s stress resistance network [66]. For instance, GST effectively neutralizes reactive oxygen species generated in plants post-stress, while its overexpression bolsters plant resilience to abiotic stress. Notably, two genes (SORBI_3001G249500, SORBI_3001G249600) showed significant downregulation, suggesting their potential negative regulation. Despite extensive evidence highlighting *GSTs* upregulation in enhancing plant stress resistance, research on its downregulation remains limited, warranting further exploration. Furthermore, the gene (SORBI_3001G318800) displayed significant upregulation consistently across all periods, indicating its robust response to saline–alkali stress. Divergent metabolic pathways distinguish the aboveground and underground sections. Post saline–alkali stress, activation of stress response genes in the aboveground section triggers signaling pathways, amplifying plant resilience to stress conditions [67] (Figure 12; Tables S11–S14). For example, MAPK functions in plant tolerance against biological and non-biological stresses, and H_2_O_2_ plays a role in the post-oxidative stress response [68,69]. The study observed the induction of a gene encoding serine/threonine protein kinase OXI1, vital for downstream pathway activation during H_2_O_2_ production [70]. Additionally, upregulation of SORBI_3010G274500, encoding CAT, and SORBI_3007G177000, encoding APX, implies their role in shielding cells from stress damage and enhancing sorghum’s saline–alkaline tolerance. In rice, overexpression of *OsAPXa* or *OsAPXb* enhances plant salt tolerance [71].

Furthermore, the ‘sucrose and starch metabolism’ pathway exhibited significant enrichment under stress [72]. Trehalose serves as the plant’s stress energy source. However, our study revealed a decrease in *TPP1* (SORBI_3004G293500) and *TPP6* (SORBI_3009G200200) expression in buds with prolonged stress, suggesting saline–alkali stress inhibits *TPP* expression, reducing trehalose content and consequently altering sorghum’s ‘starch and sucrose metabolism’ in its aboveground parts. In contrast, *TPP2* (SORBI_3001G303900) expression, responsible for trehalose biosynthesis, showed an upward trend in roots. This discrepancy suggests varied stress intensities prompt distinct resistance mechanisms in the aboveground and underground parts under saline–alkali stress.

Additionally, various transcription factor families in plants play major roles in salt tolerance by regulating the transcription of downstream genes (Figure 8). Notably, WRKY, AP2/ERF-ERF, C2H2, MYB, bHLH and NAC are among the transcription factor families involved in this regulation [73]. The AP2/ERF-ERF family is a unique gene family that regulates plant stress responses by binding to cis-elements in the promoter region of stress-responsive genes. For instance, OsSERF1 (salt-responsive ERF1) is a hub regulator of salt stress in rice [74]. Overexpression of *HvDREB1* in barley enhances its resistance to high salt stress [75]. Similarly, overexpression of TaERF4 in wheat improves its salt tolerance under salt stress [76]. Additionally, *GmERF* isolated from soybean enhances salt and drought resistance in transgenic tobacco [77]. In our study, we detected a total of 127 differentially expressed genes encoding AP2/ERF-ERF proteins, while the role of the WRKY family in response to environmental stress has also received widespread attention [78]. Overexpression of *AtWRKY61* in transgenic Arabidopsis may respond to abiotic stresses by interacting with *AtWRKY9* [79], and overexpression of *WRKY3* in tobacco enhances plant salt tolerance [80]. In our study, *SbWRKY61* (SORBI_3010G035300) and *SbWRKY3* (SORBI_3008G107500) exhibited a co-upregulation trend consistent with previous research findings. These results suggest the potential regulatory functions of the predicted transcription factor families in sorghum’s response to saline–alkaline stress.

**Figure 13:**
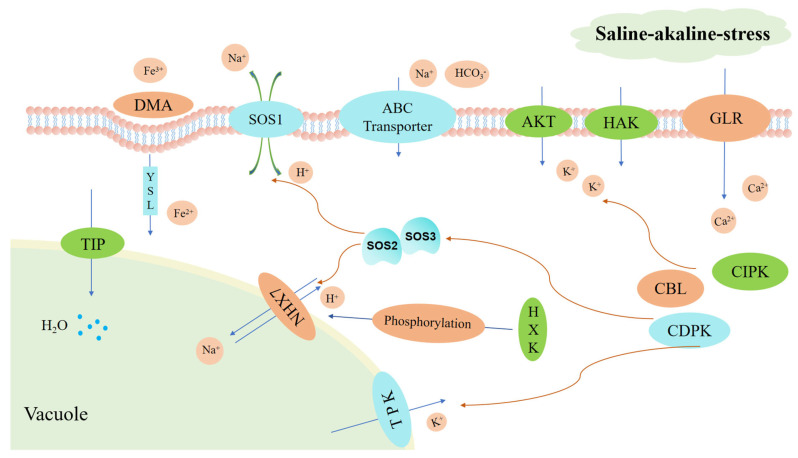
Saline–alkaline stress response model of *Sorghum bicolor* (L.).

In addition, the ratio of K^+^ to Na^+^ in the cytoplasm should be maintained at a high level, and these ions’ homeostasis can ensure the survival of plants [81]. However, under saline–alkaline stress, excessive influx of Na^+^ and OH^−^ disrupts the selective absorption of ions by plants, thus disturbing K^+^/Na^+^ homeostasis in plant cells [23] (Figure 13). This imbalance in the K^+^/Na^+^ ratio in plants triggers calcium, acting as a second messenger, to mediate the stress response [82]. Upregulated genes encoding CBL, CIPK and CDPK help regulate Ca^2+^ homeostasis [83]. The interaction between CBL and CIPK facilitates the activation of high-affinity potassium transporters (HAK) via phosphorylation, which enhances potassium ion absorption and preserves a high K^+^/Na^+^ ratio [84]. Stress-induced CDPK phosphorylates TPK in vacuoles, inhibiting cytoplasmic K^+^ influx [85,86]. Increasing K^+^ levels in plants can moderately regulate salt tolerance. In sorghum, most of the genes encoding these proteins are also upregulated, suggesting a shared regulatory mechanism. Moreover, under high pH conditions, HCO_3_^−^ hampers iron transfer, leading to iron deficiency in plants, severely affecting photosynthesis and inhibiting plant growth and yield. Monocotyledonous gramineous plants often employ chelation strategies to transport iron ions, such as secreting siderophore 2′-deoxymugineic acid (DMA) in the rhizosphere to chelate Fe^3+^ in the surrounding soil and transport it into plant cells through yellow stripe protein (YSL) [87]. Fe^2+^ is subsequently transported to various organelles and tissues by other proteins [88]. In this study, upregulated genes encoding *DMA* (SORBI_3008G109300) and *YSL1* (SORBI_3004G299600) were observed in roots, indicating that sorghum may increase iron ion absorption through this pathway. Additionally, under saline–alkaline stress, nitrogen metabolism and absorption are disrupted by limited NO_3_^−^ transport. Plant roots transport NH_4_^+^ through the ammonium transporter protein family (AMT) and NO_3_^−^ through the high-affinity nitrate transporter protein family (NRT) to compensate for the lack of content in roots [89,90]. In sorghum roots, we observed the upregulation of a gene encoding *AMT* (SORBI_3004G173200) and three genes encoding NRT (SORBI_3004G009200, SORBI_3004G009400 and SORBI_3004G009500), suggesting that sorghum may actively adapt to the saline–alkaline environment by upregulating these genes. Furthermore, the salt overly sensitive (SOS) pathway responds to saline–alkaline stress by facilitating Na^+^ efflux and compartmentalization to prevent excessive Na^+^ accumulation in the cytoplasm and subsequent damage to plants [91,92]. This study identified an upregulated gene (SORBI_3009G069800) encoding hexokinase 7, which phosphorylates the Na^+^/H^+^ antiporter (*NHX*). Previous research found that *NHX* plays a vital role in increasing Na^+^ compartmentalization to vacuoles and/or controlling long-distance Na^+^ transport, which participates in an important component of the SOS pathway in plants [93,94,95,96] (Figure 13). These findings suggest that the identified regulatory elements may contribute greatly to the response to saline–alkaline stress in sorghum.

## 4. Materials and Methods

### 4.1. Plant Culture and Treatments

A 2% sodium hypochlorite solution was used to disinfect sorghum seeds for 8 min, and distilled water was used to rinse the sorghum seeds 3 times. Then, we wrapped the seeds in filter paper and put them in light incubators for seeding. The temperature was set to 23 °C, and the light regime was 16 h light and 8 h dark every day. When the seeds sprouted to 30–50 mm, the seedlings were transferred to hydroponics boxes and cultured with half-strength modified Hoagland nutrient solution, which was changed every third day. When the stage of two leaves and one heart appeared, uniformly sized seedlings were selected for treatment and divided into a control group and a NaHCO_3_ treatment group. The control group was planted with the same solution as before; the other group was managed by 150 mM NaHCO_3_ in modified Hoagland solution, and its pH was set to 8.0. The aboveground parts and roots of the CK (0 h) and the treated group (NaHCO_3_ treatment for 6, 12 and 24 h) were randomly collected. We obtained 24 samples in total, which were collected by rapid freezing and then saved in the fridge at −80 °C. The portion of the plant situated above the seed, encompassing the stems and leaves, is designated the aboveground section.

### 4.2. Determination of Antioxidant Enzyme Activities, Proline and Malondialdehyde Concentrations

The antioxidant enzymes included superoxide dismutase (SOD), ascorbate peroxidase (APX) and peroxidase (POD). Meanwhile, the contents of the nonenzymatic antioxidants proline (PRO) and malondialdehyde (MDA) were determined. All were quantified using a detection kit (Solarbio, Beijing, China), which contained the details of the experimental procedure. Approximately 100 mg of fresh tissue was sampled for each trial and each experiment was carried out for three replicates individually [97].

### 4.3. RNA Extraction and Transcriptome Sequencing

Following the process, the EASY spin kit (Ebbend) (Aidlab, Beijing, China) was used in the extraction of total RNA from the aboveground portion and the roots. A NanoDrop 2000 spectrophotometer (NanoDrop, Wilmington, DL, USA), which is a professional platform to quantify the samples, was initially used to quantify the RNA concentration. Then, Agilent 2100/4200 (Agilent, Santa Clara, CA, USA) was used to interpret the RNA sample quality and accurately quantify the concentration. The RNA was purified, and a cDNA library was constructed and programmed according to previously published methods. When the construction of the library was finished, the Qubit 3.0 instrument (Thermo Fisher Scientific, Carlsbad, CA, USA) was available and was used for initial quantification. Illumina NovaSeq 6000 (Illumina, San Diego, CA, USA) is a mature sequencing technology and was used for transcriptome sequencing in this study after library inspection was qualified.

### 4.4. Transcriptome Data Quality Control and Assembly

All raw reads obtained by sequencing were filtered as follows: reads with an N number > 3 in the single-ended sequencing reads were eliminated; reads with a mass value of less than 5 base ratio ≥ 20% were removed; reads containing adapters were removed and strictly filtered to gather clean data for follow-up analysis.

### 4.5. Analysis of Functional Enrichment of DEGs

The differential expression of genes was analyzed with edgeR3.3.3 and the *p* value and *p*-adj values of differential expression were calculated. To control the false-positive rate, the differential genes were also combined with fold change to screen, with |log2 (fold change)| > 1.0; a q value < 0.05 was used as the criterion to screen the DEGs. Top GO v 3.14 is a practical software package for DEG analyses such as GO annotation, which was used in this study. Subsequently, KOBAS v3.0 software was applied to enrich the DEGs from the Kyoto Encyclopedia of Genes and Genomes (KEGG), and we chose the top 20 essentially enriched pathways to perform statistical analysis (In certain instances, when the number of significantly enriched pathways was less than 20, we included additional pathways that were not significantly enriched to complete the list, achieving a total of 20 pathways). Here, the essential enrichment pathway would take control of the most significant biochemical metabolic pathways and signal transduction pathways contained in the genes.

### 4.6. Weighted Gene Co-Expression Network Analysis (WGCNA)

The gene expression matrix utilized in this research originated from gene expression during salt–alkali stress at various time points. Employing the BMK Cloud platform (http://www.biocloud.net, accessed on 11 April 2023), the WGCNA network was formulated using a fold threshold of 0.5 and a minimum gene count of 30, thereby consolidating closely associated genes into distinct modules.

### 4.7. Construction of the Protein–Protein Interaction (PPI) Network

The protein–protein interaction (PPI) network serves to elucidate the cellular functionalities of proteins within organisms and aids in pinpointing pivotal proteins associated with salt–alkali stress. Utilizing the STRING database (http://string-db.org/, accessed on 14 April 2023), highly positively correlated modules, as identified by WGCNA analysis among differentially expressed genes, were leveraged to establish PPI relationships. Following this, a network graph was generated via visualization using Cytoscape v3.7.1 [98]. The primary nodes within the PPI network involved in protein interactions are regarded as hub proteins.

### 4.8. Identification of Transcription Factors

The prediction of transcription factors was managed by iTAK 1.6 online (http://itak.feilab.net/cgi-bin/itak/online_itak.cgi, accessed on 11 May 2023) for all DEGs in the transcriptome data.

### 4.9. Validation using Real-Time Quantitative PCR (RT-qPCR)

We picked 10 differentially expressed genes which would back up the reliability of the RNA-seq data, including five upregulated genes and five downregulated genes. The BIOER FQD-48A system (BIOER, Hangzhou, China) was usually utilized for RT-qPCR. The gene primer sequences used are in the attached table, and actin 1 was selected as the internal reference gene (Table S10). Regression analysis was performed using SPSS v25; the 2^−ΔΔCt^ method which was reported previously was employed to estimate gene expression [99]. Every treatment group was subjected to the experiment 3 times.

## 5. Conclusions

For this study, we conducted transcriptome sequencing on sorghum subjected to saline–alkaline stress (150 mM NaHCO_3_, pH = 8.0). We identified 6253 co-expressed DEGs and classified them using gene ontology (GO) and KEGG pathways. The findings revealed that the DEGs were primarily involved in phenylpropanoid biosynthesis, phytohormone signal transduction and starch and sucrose metabolism. Additionally, we discovered numerous transcription factors (TFs) associated with saline–alkaline tolerance and identified several key DEGs involved in cellular ion absorption and transporters, including *DMA* (SORBI_3008G109300), *YSL1* (SORBI_3004G299600) and *WRKY61* (SORBI_3010G035300). These results provide valuable insights into the molecular and physiological regulatory processes underlying salt tolerance in sorghum and offer useful knowledge for enhancing the yield and quality of sorghum in saline–alkali land.

## Figures and Tables

**Table 1:** Transcription factors with large differentially expressed folds.

Gene Family	Gene Name	Average log2 (Fold Change)	TF Id
AP2/ERF-ERF	SORBI_3002G069400	5.628355581	Sobic.002G069400.1.p
	SORBI_3005G103000	5.185617358	Sobic.005G103000.1.p
	SORBI_3007G205100	4.154163003	Sobic.007G205100.1.p
	SORBI_3002G269600	3.383162754	Sobic.002G269600.1.p
	SORBI_3007G162700	3.217188466	Sobic.007G162700.1.p
BHLH	SORBI_3009G057000	6.300233713	Sobic.009G057000.3.p
	SORBI_3003G077100	6.164343331	Sobic.003G077100.1.p
	SORBI_3008G153801	6.129472986	Sobic.008G153801.1.p
	SORBI_3002G267000	5.565088837	Sobic.002G267000.1.p
	SORBI_3007G008500	5.507180988	Sobic.007G008500.3.p
MYB	SORBI_3001G056000	4.934458789	Sobic.001G056000.1.p
	SORBI_3001G158600	3.955308363	Sobic.001G158600.2.p
	SORBI_3007G177100	2.598003641	Sobic.007G177100.1.p
	SORBI_3003G034500	2.531600604	Sobic.003G034500.1.p
	SORBI_3005G155900	2.451087788	Sobic.005G155900.1.p
NAC	SORBI_3006G141900	7.874140412	Sobic.006G141900.1.p
	SORBI_3001G018350	5.66101195	Sobic.001G018350.1.p
	SORBI_3003G333300	5.361257257	Sobic.003G333300.1.p
	SORBI_3005G028500	4.949789354	Sobic.005G028500.1.p
	SORBI_3002G200300	4.380423241	Sobic.002G200300.1.p
WRKY	SORBI_3005G014200	7.17739049	Sobic.005G014200.1.p
	SORBI_3010G148800	5.826129024	Sobic.010G148800.1.p
	SORBI_3008G029200	4.578702823	Sobic.008G029200.1.p
	SORBI_3009G247700	2.276355842	Sobic.009G247700.1.p
	SORBI_3007G217700	2.037259465	Sobic.007G217700.6.p
C2H2	SORBI_3003G242300	7.142275339	Sobic.003G242300.2.p
	SORBI_3005G025100	6.269975035	Sobic.005G025100.1.p
	SORBI_3003G242400	5.907308008	Sobic.003G242400.2.p
	SORBI_3004G066700	3.077041905	Sobic.004G066700.1.p
	SORBI_3005G225700	2.760863094	Sobic.005G225700.1.p

## Data Availability

The RNA-seq reads are available under Bio Project PRJNA1000939 in the NCBISRA database under accession SUB13694414.

## Supplementary Materials

The following supporting information can be downloaded at: https://www.mdpi.com/article/10.3390/ijms242316831/s1.

## Abbreviations

CDPK	Calcium-dependent protein kinase
CBL	Calcineurin B-like protein
CIPK	CBL-interacting protein kinase
KT	Potassium transporter
NHX	Na^+^/H^+^ antiporter
AMT	Ammonium transporter
NRT	Nitrate transporter
WGCNA	Weighted gene co-expression network analysis
PPI	Protein–protein interaction
GO	Gene ontology
KEGG	Kyoto Encyclopedia of Genes and Genomes
