# Supplementary material for: Co-Expression Network Analysis of the Transcriptome Identified Hub Genes and Pathways Responding to Saline–Alkaline Stress in Sorghum bicolor L
Source: Int J Mol Sci. 2023 Nov 27;24(23):16831. doi: 10.3390/ijms242316831 (PMC10706439; doi:10.3390/ijms242316831)
Supplement: Supplementary file 1 [file ijms-24-16831-s001.zip › ijms-2614774-supplementary.pdf]

Table S1. Growth of shoots and root lengths.

		0 h		6 h		12 h		24 h	
		**CK**	**EG**	**CK**	**EG**	**CK**	**EG**	**CK**	**EG**
1	shoot	98	94	103	95	105	95	105	97
	root	83	85	84	87	86	88	85	88
2	shoot	103	104	104	111	108	111	110	112
	root	87	87	87	89	89	91	90	91
3	shoot	100	102	102	102	102	104	103	105
	root	86	83	87	84	88	85	88	84
4	shoot	104	99	105	100	106	101	106	101
	root	92	83	92	84	92	86	92	87
5	shoot	109	95	109	96	111	97	112	97
	root	81	84	82	86	83	88	82	89
6	shoot	103	97	104	98	105	98	105	99
	root	82	83	82	84	83	84	84	86

Table S1: In the table, CK stands for control check , EG stands for Processing Group.

Table S2. Comparison of significance of shoots and root growth.

	shoot		root	
	**CK**	**EG**	**CK**	**EG**
0 h	102.8±3.8	98.5±3.9	85.2±4.1	84.2±1.6
6 h	104.5±2.4	100.3±5.8	85.7±3.8	85.7±2.1
12 h	106.2±3.1	101.0±5.8	86.3±3.8	86.7±2.8
24 h	106.8±3.4	101.8±5.8	86.8±3.8	87.5±2.4

Table S2: In the table, CK stands for control check , EG stands for Processing Group

Table S3. GO analysis was performed on the detected co-expression modules.

Category	Term	Count
GOTERM_BP_DIRECT	GO:0050896~response to stimulus	29
GOTERM_BP_DIRECT	GO:0006749~glutathione metabolic process	25
GOTERM_BP_DIRECT	GO:0009664~plant-type cell wall organization	13
GOTERM_BP_DIRECT	GO:0009738~abscisic acid-activated signaling pathway	13
GOTERM_BP_DIRECT	GO:0019941~modification-dependent protein catabolic process	8
GOTERM_CC_DIRECT	GO:0005886~plasma membrane	105
GOTERM_CC_DIRECT	GO:0005576~extracellular region	43
GOTERM_CC_DIRECT	GO:0048046~apoplast	24
GOTERM_CC_DIRECT	GO:0016020~membrane	69
GOTERM_CC_DIRECT	GO:0005634~nucleus	202
GOTERM_MF_DIRECT	GO:0004364~glutathione transferase activity	25
GOTERM_MF_DIRECT	GO:0043565~sequence-specific DNA binding	55
GOTERM_MF_DIRECT	GO:0003700~transcription factor activity, sequence-specific DNA binding	94
GOTERM_MF_DIRECT	GO:0005506~iron ion binding	53
GOTERM_MF_DIRECT	GO:0004497~monooxygenase activity	38

Table S3: Gene ontology (GO) was used to perform functional enrichment analysis of up-regulated genes in the detected co-expression module. It is divided into three parts : Molecular Function (MF) , Biological Process (BP) , and Cell Component (CC).

Table S4. KEGG analysis was performed on the detected co-expression modules

Category	Term	Count
KEGG_PATHWAY	sbi01110:Biosynthesis of secondary metabolites	124
KEGG_PATHWAY	sbi01100:Metabolic pathways	186
KEGG_PATHWAY	sbi00480:Glutathione metabolism	22
KEGG_PATHWAY	sbi04075:Plant hormone signal transduction	30
KEGG_PATHWAY	sbi00592:alpha-Linolenic acid metabolism	11
KEGG_PATHWAY	sbi04016:MAPK signaling pathway - plant	35
KEGG_PATHWAY	sbi00960:Tropane, piperidine and pyridine alkaloid biosynthesis	8
KEGG_PATHWAY	sbi02010:ABC transporters	11
KEGG_PATHWAY	sbi00999:Biosynthesis of various plant secondary metabolites	10
KEGG_PATHWAY	sbi00071:Fatty acid degradation	8
KEGG_PATHWAY	sbi00130:Ubiquinone and other terpenoid-quinone biosynthesis	8
KEGG_PATHWAY	sbi00941:Flavonoid biosynthesis	8
KEGG_PATHWAY	sbi00290:Valine, leucine and isoleucine biosynthesis	4
KEGG_PATHWAY	sbi00500:Starch and sucrose metabolism	41
KEGG_PATHWAY	sbi00940:Phenylpropanoid biosynthesis	69
KEGG_PATHWAY	sbi00350:Tyrosine metabolism	6
KEGG_PATHWAY	sbi00620:Pyruvate metabolism	9
KEGG_PATHWAY	sbi00906:Carotenoid biosynthesis	4
KEGG_PATHWAY	sbi00600:Sphingolipid metabolism	5
KEGG_PATHWAY	sbi00410:beta-Alanine metabolism	5

Table S4 : Kyoto Encyclopedia of Genes and Genomes (KEGG) was used to perform functional enrichment analysis of up-regulated genes in the detected co-expression module.

Table S5. Key genes screened by protein interaction network analysis.

Name	Probable Encoded Protein
SORBI_3006G014700	PKS_ER domain-containing protein.
SORBI_3005G119900	FAD-binding PCMH-type domain-containing protein; Belongs to the oxygen-dependent FAD-linked oxidoreductase family.
SORBI_3008G113600	Glutathione synthetase.
SORBI_3007G213700	Aminotran_5 domain-containing protein.
SORBI_3006G165400	Glutamate dehydrogenase; Belongs to the Glu/Leu/Phe/Val dehydrogenases family.
SORBI_3001G406800	Asparagine synthetase [glutamine-hydrolyzing].
SORBI_3005G003200	Asparagine synthetase [glutamine-hydrolyzing].
SORBI_3006G095700	Glutamate decarboxylase; Belongs to the group II decarboxylase family.
SORBI_3001G294400	Protein kinase domain-containing protein; Belongs to the protein kinase superfamily.
SORBI_3001G009900	Uncharacterized protein.
SORBI_3007G153900	Nitrate reductase; Nitrate reductase is a key enzyme involved in the first step of nitrate assimilation in plants, fungi and bacteria.
SORBI_3006G083000	Protein kinase domain-containing protein; Belongs to the protein kinase superfamily.
SORBI_3009G172500	Uncharacterized protein; Belongs to the class-III pyridoxal-phosphate-dependent aminotransferase family.
SORBI_3001G488200	Uncharacterized protein; Belongs to the class-III pyridoxal-phosphate-dependent aminotransferase family.
SORBI_3002G304000	CN hydrolase domain-containing protein.
SORBI_3006G014700	PKS_ER domain-containing protein.

Table S6. DEGs involved in the plant hormone biosynthesis and the plant hormone signal transduction in shoots.

**KEGG**	**GENE ID**	**Description**	**log$_2$(fold change)**		
			T6s vs CKs	T12s vs CKs	T24s vs CKs
AUX1	SORBI_3001G439000	auxin transporter-like protein 2	-3.446892786	-2.555374354	-1.185243466
AUX/IAA	SORBI_3003G291200	auxin-responsive protein IAA6-like	2.008256866	1.30104417	2.242942155
	SORBI_3005G098400	auxin-responsive protein IAA27	-4.714654434	-2.743843539	-3.44036086
GH3	SORBI_3008G015100	probable indole-3-acetic acid-amido synthetase GH3.8	1.66009611	1.725944624	1.404131982
	SORBI_3003G015000	jasmonic acid-amido synthetase JAR2	1.306718684	1.501367974	1.648489596
SAUR	SORBI_3010G027700	auxin-responsive protein SAUR50-like	-3.543858876	-2.047168021	-1.788338123
	SORBI_3004G156300	SAUR family protein	-3.756682887	-5.641549997	-5.462083002
	SORBI_3010G252500	auxin-induced protein 6B	2.113892573	1.97972386	4.255872578
	SORBI_3004G302200	SAUR family protein	-3.99802406	-3.898316724	-2.04996782
	SORBI_3006G252466	indole-3-acetic acid-induced protein ARG7	-3.061306896	-2.096205086	-1.470979
PYR/PYL	SORBI_3004G113800	abscisic acid receptor PYL8	1.572315235	1.30362038	1.135176639
	SORBI_3001G403300	abscisic acid receptor PYL4	-1.76808841	-1.942006437	-1.468673074
PP2C	SORBI_3009G213000	probable protein phosphatase 2C 50	1.956911628	1.990065372	1.206510433
	SORBI_3006G018400	probable protein phosphatase 2C 37	4.177362155	4.161886732	4.381808801
SNRK2	SORBI_3001G294400	serine/threonine-protein kinase SAPK3	1.622914902	2.113384344	1.780231248
ABF	SORBI_3010G081800	bZIP transcription factor 23	1.216711367	2.258064675	1.18790428
ETR	SORBI_3004G349175	ethylene receptor 3	1.276866772	1.374318552	1.791287168
EBF1/2	SORBI_3010G186200	EIN3-binding F-box protein 1	1.804282625	1.257362928	1.070673081
ERF1/2	SORBI_3001G002500	ethylene-responsive transcription factor 1B	4.099297818	3.375950755	5.07620533

Table S7. DEGs involved in the plant hormone biosynthesis and the plant hormone signal transduction in roots.

KEGG	GENE ID	Description	log2(fold change)		
			T6r vs CKr	T12r vs CKr	T24r vs CKr
AUX1	SORBI_3001G439000	auxin transporter-like protein 2	-2.046680977	-2.333959431	-2.264297362
	SORBI_3005G052500	auxin transporter-like protein 3	-1.930960004	-3.220158278	-3.758347917
	SORBI_3001G267100	auxin transporter-like protein 3	-3.033597664	-2.224931677	-2.585844143
AUX/IAA	SORBI_3003G291200	auxin-responsive protein IAA6-like	2.67244505	3.088837986	2.635677805
	SORBI_3009G069700	auxin-responsive protein IAA16	-3.448069993	-1.022414384	-1.251701673
	SORBI_3001G161500	auxin-responsive protein IAA12	-1.207066302	-2.480032918	-1.859806304
	SORBI_3010G180600	auxin-responsive protein IAA23	-2.333237399	-2.518437115	-1.3139511
ARF	SORBI_3006G255300	auxin response factor 3-like	-2.613057911	-2.406772163	-3.995893509
GH3	SORBI_3008G015100	probable indole-3-acetic acid-amido synthetase GH3.8	2.233698069	2.494488603	3.779695053
	SORBI_3003G306500	probable indole-3-acetic acid-amido synthetase GH3.2	2.395054601	3.8995514	6.757989764
	SORBI_3003G015000	jasmonic acid-amido synthetase JAR2	2.007602313	2.023791198	2.148718899
	SORBI_3009G187700	probable indole-3-acetic acid-amido synthetase GH3.4	-2.810719992	-2.878627439	-2.298571854
SAUR	SORBI_3010G252500	auxin-induced protein 6B	2.038103676	2.519959086	3.780109674
	SORBI_3006G216400	auxin-responsive protein SAUR71	2.650882554	2.346329686	2.743188107
	SORBI_3002G213600	SAUR family protein	2.065083887	1.022819077	1.15546743
PP2C	SORBI_3009G213000	probable protein phosphatase 2C 50	1.58230168	1.358931987	3.117165003
	SORBI_3009G238600	probable protein phosphatase 2C 51	2.147395906	2.669900207	2.745827534
SNRK2	SORBI_3003G370100	serine/threonine-protein kinase SAPK4	1.255908861	1.247231066	1.941659935
	SORBI_3006G083000	serine/threonine-protein kinase SAPK7	-1.391020809	-1.176003888	-1.619472781
	SORBI_3001G350700	serine/threonine-protein kinase SAPK1	1.525242263	1.601703166	2.088073557
ABF	SORBI_3002G225100	bZIP transcription factor TRAB1	1.265075511	1.296856225	3.303029477
ETR	SORBI_3009G050400	probable ethylene response sensor 2	1.366117113	1.862481233	1.703423301
CTR1	SORBI_3002G133400	serine/threonine-protein kinase CTR1	1.120957075	1.04275212	2.387459164
EIN3	SORBI_3004G189100	putative ETHYLENE INSENSITIVE 3-like 4 protein	1.303950753	1.605420615	2.636172318

Table S8. DEGs in ion transport pathways in shoots cells.

GENE ID	Description
SORBI_3006G213500	probable potassium transporter 11 isoform X1 [Sorghum bicolor]
SORBI_3010G112800	potassium transporter 24 [Sorghum bicolor]
SORBI_3010G197500	potassium transporter 10 isoform X1 [Sorghum bicolor]
SORBI_3001G132800	calcium-dependent protein kinase 9 [Sorghum bicolor]
SORBI_3004G025000	calcium-dependent protein kinase 4 isoform X2 [Sorghum bicolor]
SORBI_3005G024200	CBL-interacting protein kinase 32 [Sorghum bicolor]
SORBI_3009G034200	CBL-interacting protein kinase 17 [Sorghum bicolor]
SORBI_3009G097300	CBL-interacting protein kinase 22 [Sorghum bicolor]
SORBI_3009G195800	CBL-interacting protein kinase 19 [Sorghum bicolor]
SORBI_3003G139500	CBL-interacting protein kinase 1 [Sorghum bicolor]
SORBI_3006G105600	ABC transporter B family member 19 [Sorghum bicolor]
SORBI_3007G164000	ABC transporter F family member 1 [Sorghum bicolor]
SORBI_3009G022000	ABC transporter G family member 16 [Sorghum bicolor]
SORBI_3009G058100	ABC transporter F family member 4 [Sorghum bicolor]
SORBI_3001G499100	ABC transporter G family member 22 isoform X1 [Sorghum bicolor]
SORBI_3003G267300	ABC transporter B family member 11 [Sorghum bicolor]
SORBI_3002G162400	two pore potassium channel c [Sorghum bicolor]
SORBI_3004G013300	glutamate receptor 3.1 [Sorghum bicolor]
SORBI_3001G195800	aquaporin NIP3-1 [Sorghum bicolor]
SORBI_3001G208500	aquaporin TIP3-1 [Sorghum bicolor]
SORBI_3003G237900	potassium channel AKT1 isoform X1 [Sorghum bicolor]

Table S9. DEGs in ion transport pathways in roots cells.

GENE ID	Description
SORBI_3001G183700	potassium transporter 21 [Sorghum bicolor]
SORBI_3001G184100	probable potassium transporter 16 isoform X1 [Sorghum bicolor]
SORBI_3002G130800	potassium transporter 18 isoform X1 [Sorghum bicolor]
SORBI_3002G417500	probable potassium transporter 9 [Sorghum bicolor]
SORBI_3007G075100	putative potassium transporter 12 isoform X1 [Sorghum bicolor]
SORBI_3010G112800	potassium transporter 24 [Sorghum bicolor]
SORBI_3010G197500	potassium transporter 10 isoform X1 [Sorghum bicolor]
SORBI_3004G279100	calcium-dependent protein kinase 5 [Sorghum bicolor]
SORBI_3003G196400	calcineurin B-like protein 9 [Sorghum bicolor]
SORBI_3003G208400	calcineurin B-like protein 5 isoform X2 [Sorghum bicolor]
SORBI_3007G140900	CBL-interacting protein kinase 6 [Sorghum bicolor]
SORBI_3008G032000	CBL-interacting protein kinase 15 [Sorghum bicolor]
SORBI_3009G034200	CBL-interacting protein kinase 17 [Sorghum bicolor]
SORBI_3010G186300	CBL-interacting protein kinase 24 [Sorghum bicolor]
SORBI_3001G379600	CBL-interacting protein kinase 10 [Sorghum bicolor]
SORBI_3001G390200	CBL-interacting protein kinase 31 isoform X1 [Sorghum bicolor]
SORBI_3002G424500	CBL-interacting protein kinase 31 [Sorghum bicolor]
SORBI_3003G024600	putative CBL-interacting protein kinase 13 [Sorghum bicolor]
SORBI_3003G302800	CBL-interacting protein kinase 19 [Sorghum bicolor]
SORBI_3001G383300	ABC transporter B family member 25, mitochondrial [Sorghum bicolor]
SORBI_3002G054300	ABC transporter F family member 4 [Sorghum bicolor]
SORBI_3003G177350	putative ABC transporter C family member 15 isoform X1 [Sorghum bicolor]
SORBI_3003G180400	ABC transporter B family member 11 [Sorghum bicolor]
SORBI_3003G215600	ABC transporter G family member 35 [Sorghum bicolor]
SORBI_3003G267300	ABC transporter B family member 11 [Sorghum bicolor]
SORBI_3003G267700	ABC transporter B family member 4 [Sorghum bicolor]
SORBI_3003G387300	ABC transporter G family member 5 [Sorghum bicolor]
SORBI_3003G398100	ABC transporter B family member 26, chloroplastic-like [Sorghum bicolor]
SORBI_3004G087800	ABC transporter G family member 39 [Sorghum bicolor]
SORBI_3004G128100	ABC transporter E family member 2 isoform X1 [Sorghum bicolor]
SORBI_3005G061000	ABC transporter G family member 25 [Sorghum bicolor]
SORBI_3006G022200	ABC transporter C family member 4 [Sorghum bicolor]
SORBI_3006G114100	ABC transporter G family member 51 [Sorghum bicolor]
SORBI_3006G195800	putative ABC transporter C family member 15 [Sorghum bicolor]
SORBI_3007G120000	ABC transporter A family member 7 [Sorghum bicolor]
SORBI_3007G164000	ABC transporter F family member 1 [Sorghum bicolor]
SORBI_3010G169000	ABC transporter C family member 8 [Sorghum bicolor]
SORBI_3008G109300	2'-deoxymugineic-acid 2'-dioxygenase-like [Sorghum bicolor]
SORBI_3004G299600	iron-phytosiderophore transporter yellow stripe 1 isoform X1 [Sorghum bicolor]
SORBI_3002G162400	two pore potassium channel c [Sorghum bicolor]

SORBI_3002G209100	glutamate receptor 2.8 isoform X2 [Sorghum bicolor]
SORBI_3004G324000	glutamate receptor 2.8 [Sorghum bicolor]
SORBI_3010G092600	aquaporin NIP2-2 [Sorghum bicolor]
SORBI_3010G146100	probable aquaporin TIP2-2 [Sorghum bicolor]
SORBI_3003G300600	potassium channel KAT3 isoform X1 [Sorghum bicolor]
SORBI_3003G237900	potassium channel AKT1 isoform X1 [Sorghum bicolor]
SORBI_3001G240300	cation/calcium exchanger 1 [Sorghum bicolor]
SORBI_3009G069800	hexokinase-7 [Sorghum bicolor]

Table S10. Primers used in experiment of gene expression by qPCR.

GENE ID	Forward Primer	Reverse Primer
Actin 1	TCTTCCAACCCTACCGCACCA	CGTCATCTCCAGCGAACCCAGCCTT
SORBI_3004G082600	CCCGAGGAAAATGATCGCCT	AGGCAGTCCACCCAGTAGAA
SORBI_3008G011200	CATCAAGGCCGACATCTGGT	CTTGAACGCCCTGGAGAACT
SORBI_3001G218800	CACATTGCTCAAGTGCTGGG	AGTCCAAACTGACGACCACC
SORBI_3001G350400	CCCTCAGCCTTGTGCCAATA	TGCCCATCCGCTGAAATCTT
SORBI_3009G156000	ACTCAACCAGCGATGAGGAC	TTCTAGCGCAGTGAGGTGTC
SORBI_3002G256500	TGGAGTGCAGGCTTTCAGAG	CTGGCGAGTTCGTTCCTTCT
SORBI_3004G327600	AGAAGCAGAGGCTGATCGAC	TTGGATCACCTCGTTGAGGC
SORBI_3002G027700	TTCACGGAGGAGGGATAGCA	GGGAGCCATTCTCCAACACA
SORBI_3009G189501	AAGCAATCCGTCTCGTCGTT	AGGTAGCCGAAGACAAGTGC
SORBI_3009G069800	CCTCCCAATCCAAGGTGGTC	CCTCCCAATCCAAGGTGGTC
SORBI_3002G162400	CCCATCCCCAGCCGCCTC	CGGCGGAGGAAGGGTCCT
SORBI_3007G164000	GAGGTTCCCCTTCTTCCCCC	GAGGTTCCCCTTCTTCCCCC
SORBI_3010G169000	GTCCCCGTGCGTGCAGAG	CTCACCGGCCACGAGATCG
SORBI_3002G054300	CAAGTCGTCCAAGTCGGCG	CGTCGTCGCCGGTGACGC
SORBI_3008G032000	TTGTTCATCTTCATGAGGTCATGG	TTGTTCATCTTCATGAGGTCATGG
SORBI_3001G379600	TTGTTCATCTTCATGAGGTCATGG	AAAATCCGACACCTTGAGGTTCT
SORBI_3003G196400	GATGCTGATGCCGATAAGGATG	TGACAGCCAACAGTCATCAAGG
SORBI_3002G141900	CATTCCCTCCAGACTCGGACT	CCAGCGAAGTGGGAAGCG
SORBI_3004G293500	TGATTCCGCTAGAGGACTCAAGC	TGATTCCGCTAGAGGACTCAAGC
SORBI_3010G035300	AGAGGACTACCGATCTCTTCAATTG	AGCAGCACTATTCTTGTCTTCCTTC
SORBI_3006G014700	CGACTGCGACCACTGCCG	TACACGGTGATCCCCGCG

Table S11. DEGs of Transcriptome network.

KEGG	GENE ID	Description	log2(fold change)		
			T6s vs CKs	T12s vs CKs	T24s vs CKs
Cinnamic acid→Cinnanayol-CoA	SORBI_3004G272700	probable 4-coumarate--CoA ligase 2	-2.988051392	-2.419823005	-0.402261017
Cinnamoyl CoA→Cinnamaldehyde	SORBI_3002G146000	cinnamoyl-CoA reductase 2	-0.516027994	-0.57162158	0.260162014
	SORBI_3004G065600	cinnamoyl-CoA reductase 1	-3.519446148	-3.150263622	-2.428909365
P-Coumaroyl acid→P-Coumaroyl-CoA	SORBI_3004G272700	probable 4-coumarate--CoA ligase 2	-2.988051392	-2.419823005	-0.402261017
ANP1	SORBI_3007G130000	mitogen-activated protein kinase kinase kinase NPK1	-6.510532291	-3.339352381	-4.414078787
NDPK2	SORBI_3008G123600	nucleoside diphosphate kinase 2, chloroplastic	-2.028129006	-2.188312417	-2.807083858
OXI1	SORBI_3006G128200	serine/threonine-protein kinase OXI1	-2.88290153	-2.237042328	-1.278181242
Surose→UDP-Glucose	SORBI_3001G344500	sucrose synthase 2	-3.166143728	-3.884623365	-2.102079233
UDP-glucose→Trehalose-6P	SORBI_3002G188500	probable alpha,alpha-trehalose-phosphate synthase	1.673805401	1.142422369	1.513387058
	SORBI_3009G200200	alpha,alpha-trehalose-phosphate synthase	-1.074356859	-1.81361535	-1.216001835
Trehalose-6P→Trehalose	SORBI_3004G293500	probable trehalose-phosphate phosphatase 1	-1.556138042	-1.414304471	-1.069870664
	SORBI_3002G184600	probable trehalose-phosphate phosphatase 6	-2.390498038	-2.873371589	-3.242254973
	SORBI_3009G200200	alpha,alpha-trehalose-phosphate synthase	-1.074356859	-1.81361535	-1.216001835
Surose-6P→D-Glucose-6P or D-Frucroe	SORBI_3006G070564	beta-fructofuranosidase, insoluble isoenzyme 2	-2.56613151	-4.000084786	-1.699926855

Table S12. DEGs of Transcriptome network.

KEGG	GENE ID	Description	log2(fold change)		
			T6r vs CKr	T12r vs CKr	T24r vs CKr
5-Oxoproline→L-Glutamate	SORBI_3001G322700	5-oxoprolinase [Sorghum bicolor]	1.451953689	1.375913599	1.176517464
Glycine	SORBI_3008G113600	glutathione synthetase, chloroplastic [Sorghum bicolor]	1.083640389	1.22965188	1.286041425
GSH→R-S-Glutathione	SORBI_3003G426000	probable glutathione S-transferase	2.253872443	2.080755511	2.127926438
	SORBI_3001G318600	probable glutathione S-transferase	2.157630746	2.022143431	5.084708355
	SORBI_3003G426300	probable glutathione S-transferase GSTU6	1.218335884	1.538804228	3.91036757
	SORBI_3008G079400	probable glutathione S-transferase GSTU6	2.770048375	2.412963259	2.316992289
	SORBI_3001G318800	probable glutathione S-transferase GSTU6	6.255574596	7.740129198	9.952179034
	SORBI_3005G212700	probable glutathione S-transferase GSTU6	1.71175633	1.759414451	4.648790291
	SORBI_3001G249500	uncharacterized LOC8063175	-7.044032733	-7.116561579	-4.45452126
	SORBI_3001G249600	probable glutathione S-transferase BZ2	-9.953702694	-10.02351634	-3.467308224
	SORBI_3001G066000	probable glutathione S-transferase BZ2	1.061700806	1.676326685	2.278030729
	SORBI_3009G043600	probable glutathione S-transferase GSTU6	1.183676917	1.223376578	1.665438797
	SORBI_3003G264400	glutathione S-transferase 4	2.596457099	3.012603911	4.012757183
	SORBI_3001G412700	probable glutathione S-transferase GSTU6	2.369450554	2.499811137	3.542798148
	SORBI_3001G319200	probable glutathione S-transferase GSTU1	1.844905354	1.83513546	3.617796681
	SORBI_3001G318500	probable glutathione S-transferase GSTF1	1.636064678	2.037455764	3.258552593
	SORBI_3001G318000	protein IN2-1	2.597441606	3.096916998	5.4884229
Uracil→5,6-Dihyclrouracil	SORBI_3002G215700	dihydropyrimidine dehydrogenase (NADP(+)), chloroplastic	1.65541644	2.137363852	1.882294206
N-Carbamoyl-β-alanine→β-Alanine	SORBI_3002G304000	beta-ureidopropionase	1.704934315	2.184078408	1.782591368
β-Alanine→β-Aminopropion-aldehyde	SORBI_3002G215700	aldehyde dehydrogenase family 7 member A1	1.813283572	2.115547106	3.524351038

1,3-Diamino-propane→β-Aminopropion-aldehyde	SORBI_3004G243400	primary amine oxidase 2	-2.385576936	-3.770480815	-1.490069485
	SORBI_3006G119200	copper methylamine oxidase	1.40825822	1.539272196	2.041118342
Spermidine→1,3-Diamino-propane	SORBI_3001G472000	polyamine oxidase-like	-2.807676514	-3.001832564	-2.879676896
	SORBI_3007G073100	polyamine oxidase	-1.891005584	-1.73193786	-3.280690611
Spermine→Spermidine	SORBI_3001G472000	polyamine oxidase-like	-1.891005584	-1.73193786	-3.280690611
	SORBI_3003G274600	probable polyamine oxidase 5	-5.842467796	-5.007035576	-3.648216997
	SORBI_3007G073100	polyamine oxidase	-1.891005584	-1.73193786	-3.280690611
β-Alanine→(R)-Pantothenate	SORBI_3001G009900	pantoate--beta-alanine ligase	1.425573766	2.122244643	3.377037481
3-Hydroxyisobutyryl-CoA→3-Hydroxy-propanoate	SORBI_3001G290000	3-hydroxyisobutyryl-CoA hydrolase 1	1.217072962	1.554401963	1.290075445
Acrylyl-CoA→3-Hydroxyisobutyryl-CoA	SORBI_3001G393800	probable enoyl-CoA hydratase 1, peroxisomal	1.217072962	1.554401963	1.290075445
Propionyl-CoA→Acrylyl-CoA	SORBI_3005G181000	acyl-coenzyme A oxidase 2, peroxisomal	3.583695886	3.894417883	4.490409233
	SORBI_3003G354400	acyl-coenzyme A oxidase 3, peroxisomal	2.183446723	2.076473821	3.850312006
	SORBI_3006G012800	acyl-coenzyme A oxidase 4, peroxisomal	3.074777427	2.945550391	8.656628975

Table S13. Transcription factor in shoots and roots.

KEGG	GENE ID	Description	log2(fold change)		
			**T6s vs CKs**	**T12s vs CKs**	**T24s vs CKs**
WRKY	SORBI_3001G055400	protein WRKY1 [Sorghum bicolor]	3.328562468	2.313466207	3.35726579
	SORBI_3004G271800	probable WRKY transcription factor 14 [Sorghum bicolor]	1.622596545	3.076794482	3.567027919
	SORBI_3007G077466	hypothetical protein SORBI_3007G077466 [Sorghum bicolor]	1.733194392	1.509590007	1.185091705
	SORBI_3008G028600	probable WRKY transcription factor 54 [Sorghum bicolor]	-3.72639145	-2.038496559	-2.231018358
	SORBI_3009G206800	probable WRKY transcription factor 56 [Sorghum bicolor]	-1.664541798	-1.222153951	-1.090451025
	SORBI_3001G084000	probable WRKY transcription factor 48 [Sorghum bicolor]	-3.138586528	-3.136701072	-2.475873442
MYB	SORBI_3007G177100	myb-related protein Zm38 [Sorghum bicolor]	2.287632906	3.101192878	2.773944624
	SORBI_3009G148100	myb-related protein 308 [Sorghum bicolor]	-2.116815746	-2.36609787	-1.370632085
	SORBI_3003G129000	transcription factor MYB117 [Sorghum bicolor]	1.147541014	1.000717694	2.611621737
	SORBI_3007G136500	hypothetical protein BDA96_07G146300 [Sorghum bicolor]	-2.857716476	-1.426434056	-1.720287052
	SORBI_3001G356000	myb-related protein 306-like [Sorghum bicolor]	1.427259516	1.161469739	2.840086043
	SORBI_3002G196000	protein ODORANT1 [Sorghum bicolor]	2.117629665	2.358857365	3.748659533
	SORBI_3003G034500	transcription factor RAX2 [Sorghum bicolor]	2.889582391	1.812154491	2.803142197
NAC	SORBI_3006G218300	hypothetical protein BDA96_06G238900 [Sorghum bicolor]	1.964672356	1.361738288	3.688400221
	SORBI_3006G279400	NAC domain-containing protein 30 [Sorghum bicolor]	1.08751927	1.38188795	1.52455875
	SORBI_3003G365500	NAC domain-containing protein 90 [Sorghum bicolor]	-4.140673498	-4.145408576	-3.069381313
	SORBI_3006G086000	NAC domain-containing protein 104-like [Sorghum bicolor]	1.626852439	1.652336017	1.933082985
bHLH	SORBI_3010G172800	putative transcription factor bHLH041 [Sorghum bicolor]	1.453547007	1.415858355	2.065175147
	SORBI_3003G102300	transcription factor bHLH162 [Sorghum bicolor]	1.218063037	3.444112185	1.966110422
	SORBI_3005G195400	transcription factor bHLH54 [Sorghum bicolor]	2.330231602	1.702927363	3.407893629
AP2	SORBI_3001G296300	ethylene-responsive transcription factor ERF104 [Sorghum bicolor]	1.849000775	1.454289197	2.141041251

SORBI_3002G350500	ethylene-responsive transcription factor RAP2-11-like [Sorghum bicolor]	1.458851119	1.481810437	1.859456749
SORBI_3007G188701	hypothetical protein SORBI_3007G188701 [Sorghum bicolor]	3.026476432	2.102783555	3.822434959
SORBI_3002G139300	ethylene-responsive transcription factor ERF096-like [Sorghum bicolor]	1.454668857	-1.141524515	1.68029745
SORBI_3006G247000	ethylene-responsive transcription factor ERF003 [Sorghum bicolor]	2.625835684	1.597872507	2.208843803
SORBI_3001G473900	ethylene-responsive transcription factor RAP2-13 [Sorghum bicolor]	2.428551312	2.398248676	2.402268096

Table S14. Transcription factor in shoots and roots.

KEGG	GENE ID	Description	log2(fold change)		
			T6r vs CKr	T12r vs CKr	T24r vs CKr
WRKY	SORBI_3007G217700	hypothetical protein SORBI_3007G217700 [Sorghum bicolor]	2.266140861	2.185623005	3.108475542
	SORBI_3008G107500	probable WRKY transcription factor 3 [Sorghum bicolor]	2.032184372	2.006505208	2.648253588
	SORBI_3010G035300	probable WRKY transcription factor 61 [Sorghum bicolor]	2.567117809	1.356330663	3.80972875
	SORBI_3009G206800	probable WRKY transcription factor 56 [Sorghum bicolor]	-2.206461417	-2.338343017	-3.226503623
	SORBI_3003G037400	probable WRKY transcription factor 50 isoform X1 [Sorghum bicolor]	1.153684217	1.105082011	1.265865909
	SORBI_3007G085300	hypothetical protein SORBI_3007G085300 [Sorghum bicolor]	-2.112845265	-1.387043297	1.213117559
MYB	SORBI_3004G303600	transcription factor LAF1 [Sorghum bicolor]	-2.202870472	-2.137415588	-2.517373482
	SORBI_3004G219800	transcription factor MYB4 [Sorghum bicolor]	-3.213410646	-1.624782799	1.618492935
	SORBI_3008G020300	transcription factor MYB30 [Sorghum bicolor]	-1.986271844	-1.706099154	-1.980038786
	SORBI_3003G280000	transcription factor MYB58 [Sorghum bicolor]	2.800162377	1.948801559	2.585314231
	SORBI_3003G034500	transcription factor RAX2 [Sorghum bicolor]	2.972557717	2.994184111	1.717982717
	SORBI_3007G177100	myb-related protein Zm38 [Sorghum bicolor]	1.73418671	2.256013859	3.43505087
NAC	SORBI_3001G071000	transcription factor JUNGBRUNNEN 1 [Sorghum bicolor]	1.927387063	2.075666941	3.48987267
	SORBI_3008G183900	transcription factor JUNGBRUNNEN 1 [Sorghum bicolor]	1.775617771	1.140943648	3.752331002

	SORBI_3008G057700	hypothetical protein SORBI_3008G057700 [Sorghum bicolor]	-2.293067452	-1.659412722	-2.99241448
	SORBI_3001G515800	NAC domain-containing protein 35 [Sorghum bicolor]	-3.214579597	-2.387157037	1.666105009
	SORBI_3006G147400	hypothetical protein SORBI_3006G147400 [Sorghum bicolor]	-1.82451557	-3.326452965	-3.829407495
	SORBI_3006G086000	NAC domain-containing protein 104-like [Sorghum bicolor]	2.850394196	3.278813638	3.612789057
bHLH	SORBI_3006G217800	unnamed protein product [Miscanthus lutarioriparius]	1.459236902	2.815169641	2.496218831
	SORBI_3001G447800	transcription factor bHLH74 isoform X1 [Sorghum bicolor]	-3.093254437	-3.090634176	-1.618804386
	SORBI_3009G036400	hypothetical protein SORBI_3009G036400 [Sorghum bicolor]	-1.555274006	-2.258997282	-2.396029572
	SORBI_3001G464300	transcription factor bHLH84 [Sorghum bicolor]	3.06253233	1.861981452	2.829980817
	SORBI_3003G099000	transcription factor bHLH69 [Sorghum bicolor]	1.731601684	2.280999635	2.503336412
AP2	SORBI_3010G052600	ethylene-responsive transcription factor RAP2-1 [Sorghum bicolor]	1.772556615	1.542869521	1.460645334
	SORBI_3001G481300	ethylene-responsive transcription factor 1 [Sorghum bicolor]	1.167559804	1.541073082	4.492174305
	SORBI_3007G188701	hypothetical protein SORBI_3007G188701 [Sorghum bicolor]	1.635089665	2.046607713	2.551462487
	SORBI_3002G139300	ethylene-responsive transcription factor ERF096-like [Sorghum bicolor]	-2.229557664	-1.233760979	2.68425325
